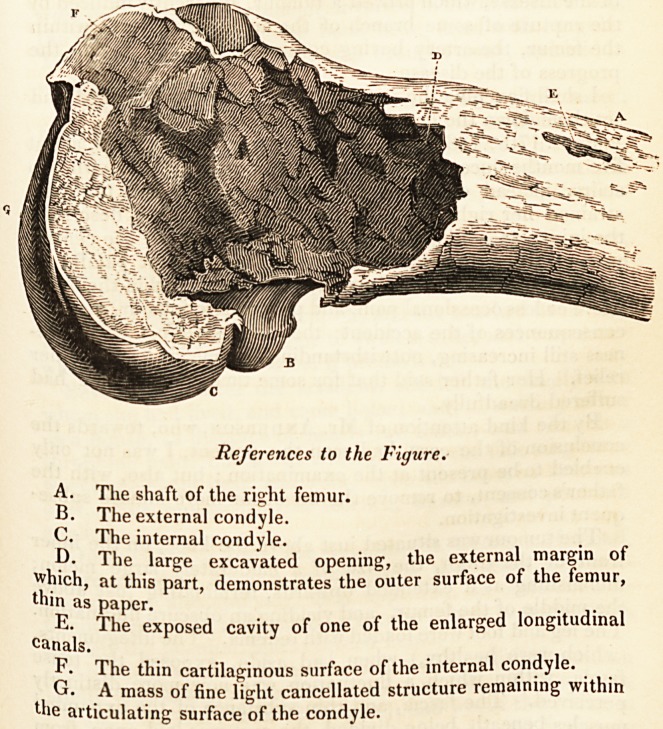# On the Causes of Some of the Symptoms Which Attend Diseases and Injuries of the Brain

**Published:** 1835-04-01

**Authors:** 


### ORIGINAL COMMUNICATIONS.

---

### *On the* Causes *of some of the* Symptoms *which attend* Diseases *and* Injuries *of the* Brain. By Mr. Mayo.

(Read before the College of Physicians.)

One of the principal objects of the science of medicine is to obtain accuracy of diagnosis. Now, accuracy of diagnosis, it is evident, cannot exist, unless the causes and mode of production of the symptoms of disease are correctly known. But the symptoms of disease are the external signs of disordered actions of the bodily organs. The inquiry into their nature is therefore a part of physiology,—*it is the physiology of disease;* and, as morbid anatomy is based upon healthy anatomy, so is the physiology of disease built upon, or elucidated by, the physiology of health. In each case the improvement of the one branch of knowledge is almost a necessary preliminary to the improvement of the other; and accordingly it has always happened that, whenever any important advance has been made in the study of healthy actions, the introduction of more just and accurate views of their pathology has followed.

The functions of the nervous system have received striking elucidation from the labours of modern physiologists: its pathology, therefore, is probably on the eve of considerable improvement. But the physiology of the nervous system has not been studied with equal success in all its parts. We remain in the same ignorance as formerly of the functions of the hemispherical masses of the enkephalon, while the offices of the medulla oblongata, of the spinal marrow, and of the nerves, have been made out with a remarkable degree of precision. This consideration may be of use in directing our pathological inquiries. It is likely that something may now be determined as to the disorders of those mental functions which are proved to have their seat in the latter parts, although it may be hopeless at present to speculate upon the symptoms of disordered actions, which we only obscurely guess to have their immediate seat in the brain.

The functions which have their physical organs in the medulla oblongata, the spinal marrow, and the nerves, are sensation, perception, the commonest instinctive impulses, and volition. The principal facts that are known upon this subject may be explained under three heads.

1. It is presumed that the mental functions enumerated are perfected in the organs above assigned to them, upon the ground that, in the higher animals, and even in the human species, those mental functions have been displayed in cases, where, either from congenital deficiency, or through removal by design or accident, the hemispherical masses of the enkephalon have been wanting. This conclusion is of such magnitude and importance, that it may appear to some present desirable to have the evidence repeated, on which it rests. A single instance, which I will select in illustration, is so complete as to have the force of an *experimentum crucis* upon the points before us: nevertheless, if it stood alone, the conclusion to which it leads is so marvellous and unexpected, that one would almost doubt the accuracy of the observations recorded in it. They are, however, borne out and confirmed beyond the reach of question by recent experiments. The instance to which I allude is contained in the history of an acephalous child, an account of which was communicated by Mr. Lawrence to the Medico-Chirurgical Society.

The child had neither cerebrum nor cerebellum; but the spinal marrow and medulla oblongata, with the nerves which rise from them, were perfect. It lived four days. During its brief existence, it manifested sensation, perception, the usual instincts of a newborn infant, and volition. It moved briskly at first; when food was put into its mouth, it swallowed it; it breathed naturally; it voided fæces, and passed urine twice on the first day, and once a day afterwards. This child gave evidence of the same mental affections as ordinary newborn infants display. It is impossible, I think, to doubt, either that the usual mental affections of a newborn child really occurred in it, or that the parts of the nervous system, which alone this infant possessed, were sufficient for these functions; or that, being sufficient in this instance, they are sufficient for the same purposes in others. The crowning masses of the enkephalon, which this infant wanted, may be presumed to be the seat of those higher functions, which are developed in human beings as life advances.

2. The medulla oblongata and spinal marrow are an aggregate of many organs. They consist of several segments, each of which originates one or more pairs of nerves, that are distributed each to a corresponding segment of the frame, and are to a certain degree independent of the others; however, it may be necessary for the ordinary purposes of life and thought that they should be all connected and continuous. In each of these segments is the apparatus for sensation, instinct, and volition, adapted to the segment of the frame which it supplies with nerves.

In proof of the correctness of this position, I shall again bring forward a single instance; the most striking, indeed, that I have met with, but to which it would be easy to add others nearly as conclusive.

I made the following experiment upon the head of a bird, which, it is to be observed, retains a kind of vitality for a short time after its separation from the body. In the head of a decapitated pigeon, I exposed the brain, and then removed, by successive slices, the cerebrum, the cerebellum, and the medulla oblongata, leaving within the head nothing but the optic tubercles, and the second and third pairs of nerves. I then divided the optic nerves. The optic tubercles thus alone remained of the whole encephalon, and were connected to the eyes by the third nerves alone. I then repeatedly pricked the stump of the optic nerves which projected from the optic tubercles: the injury was followed each time by a sudden contraction of the pupils.

I infer from this and similar experiments, that when an impression is made upon a nerve of sense, and communicated by it to the organ in which that nerve arises, that organ, or segment of the spinal marrow or medulla oblongata, is sufficient in itself to originate the instinctive impulse, which determines along the motor nerve the influence to motion. The medulla oblongata and spinal marrow consist of many such segments, independent of each other in one sense, in another materially dependent, and fitted and intended to cooperate.

3. The nerves are media of transmission only. In reference to consciousness, they form two classes, one for sensation, the other for motion. The experimental evidence adduced by Sir Charles Bell, M. Magendie, and in part by myself, in proof of this statement, is so generally known as not to require to be repeated on this occasion.

The preceding physiological principles being admitted, I propose to apply them, with others to which it may be necessary to refer, in explaining some of the symptoms of impaired voluntary motion and sensation, which are attendant upon lesions of the brain; or I shall endeavour to answer the following questions:

1. In what manner, or through what kind of influence, does a lesion of the brain produce palsy and numbness?

2. How does it happen that a lesion of one side of the brain invariably produces palsy of the opposite side of the body?

3. Why, in total hemiplegia, is the palsy of the leg less complete, and capable of being more quickly recovered from, than the palsy of the arm?

4. Why, in a slow attack of palsy, are the weakness and numbness first felt in the hands and in the feet; the parts of the limb nearest the trunk being the last affected?

5. Why is muscular palsy more frequent than loss of sensibility, or anæsthesia?

1. *In what manner, or through what kind of influence, does a lesion of the brain produce palsy?*

There are two modes of explaining the occurrence of palsy as a consequence of cerebral lesion.

One is, to suppose that the palsy results from the withholding, or interruption, of the usual supply of nervous energy derived from the brain. Nor could this hypothesis be objected to, if the palsy were experienced in continued or deliberate action alone. It might then be plausibly argued, that as the brain, in the healthy state, is the seat of reflection and the source of deliberate action, so, when partially impaired, and in a state of lesion, it is incapable of furnishing the same quota of excitation as before. But the palsy in such cases is constant and uninterrupted; and, whether loss of sensation accompany it or not, no irritation or injury of the integuments of the palsied limb will produce the smallest muscular action; that intuitive flinching and retraction of the limb, when pinched or pricked, which occurs in animals from which the brain has been removed, does not take place in cases of simple palsy dependent upon cerebral disease. The palsy therefore involves some additional element besides the mere abstraction of cerebral power.

The additional element, so shown to exist, is taken for granted in the second hypothesis, and is supposed to be an active depressing force, which strikes with feebleness the organs of volition and sensation, in part or wholly. Upon this hypothesis, which I adopt, I am disposed to believe that, when the brain is in certain states of lesion, it *actively* paralyses, by the transmission of a lowering and deleterious influence, the medulla oblongata and the spinal marrow.

It may be asked, whether it is reasonable to suppose that the brain, when in a state of lesion, *can* originate any such depressing or palsying shock? Reasoning upon analogy, it is probable that the brain may possess this property. It is certain that, upon other organs than those of animal life, the brain *does* exert such disastrous and depressing influence. For example, in persons of a nervous temperament, sudden and overwhelming intelligence will sometimes temporarily stop the heart's action. Physical impressions, again, such as crushing a part of the brain or spinal marrow, will temporarily palsy the heart; an experiment the more conclusive, that the heart's action will continue undisturbed during the entire removal of the brain and spinal marrow, if the abstraction of these parts be made with gentleness. Is it surprising that lesion of the brain, which can palsy the heart, the action of which is not derived from the nervous system, should throw palsy upon the voluntary muscles, that in so many ways are habitually influenced through it? Such a force of depression, similar in kind, although much less in degree, mental affections alone seem to have the power of temporarily producing: the "genua labant, tremor occupat artus," the weakness of terror, is probably an instance of imperfect or temporary palsy so produced.

Upon these and other grounds, which will be presently mentioned, it appears reasonable to believe that a lesion of the brain is capable of originating a depressing force, which can strike with palsy the organs from which the nerves arise; or that palsy from cerebral disease is not caused by the interruption of an accustomed stimulus, but by the production of a new and withering influence, transmitted from thence to the origins of the nerves.

2. *How does it happen that a lesion of one side of the brain invariably produces palsy of the opposite side of the body?*

The fact is remarkable from its uniformity: how is it to be accounted for? The entire analogy of the nervous system leads us to suppose that the influences which pervade it move in the direction of the threads or filaments of which the medullary substance is composed. Anatomists therefore look with curious interest to the construction of the enkephalon, in the expectation of discovering some transposition or crossing over of nervous filaments from one side to the other, through which the crossing of the depressing influence, or palsy-shock, may be supposed to be conveyed. After the most careful research, it appears that such a transposition or decussation of nervous threads, is to be found in the medulla oblongata alone. Where the medulla oblongata joins the spinal marrow, the anterior pyramids throw their fibres downwards, in oblique decussation, each to the opposite side, in such a manner that the right anterior pyramid plunges into the centre of the left half of the spinal marrow, while the fibres of the left anterior pyramid plunge into the right half of the chord.

I concur with those who think that these decussating filaments are the channels through which the palsying influence is conveyed from a diseased cerebral hemisphere to the opposite side of the frame, upon several grounds: first, because these decussating fasciculi are the only ones which have been discovered in the enkephalon; secondly, because the position of these decussating fasciculi is exactly that which experiment and observation lead us to expect to be the place of the transposition of palsy; thirdly, because all the phenomena of hemiplegia from lesion of the opposite side of the brain, may be explained upon this supposition; fourthly, because even the remarkable cases in which partial hemiplegia of the opposite side is combined with partial palsy on the side of the cerebral lesion, admits of perfect explanation on the same hypothesis.

The most conclusive facts with which I am acquainted, to show that the crossing of palsy to the opposite side is effected in the region of the medulla oblongata, are given in a very interesting paper by Dr. Yelloly, in the second volume of the Medico-Chirurgical Transactions.

Dr. Yelloly describes an experiment upon a dog, in which Sir Astley Cooper divided the right half of the spinal marrow, at the interval between the occiput and atlas: the dog became palsied *on the injured side.* It may be inferred from this experiment, that the seat of the transmission of palsy is to be found above the atlas.

Dr. Yelloly afterwards describes a case of hemiplegia, in which it was found, after death, that a tumour, of the size of a filbert, had been imbedded in, and had made pressure upon, the right side of the annular protuberance. The hemiplegia in this case had affected the left, or *opposite side* of the body.

It may be confidently inferred from this case, that the seat of the transit of palsy is behind or below the pons Varolii; but the experiment by Sir Astley Cooper, previously mentioned, establishes that it is above the spinal chord. The two instances, taken together, render it almost certain that the place of the transit of palsy is somewhere in the medulla oblongata, or its junction with the spinal chord; and it is evident how much additional force is given to this conclusion, by the fact previously mentioned, that no decussation of nervous filaments has been found, except at the latter point.

We may now inquire whether the decussation of the anterior pyramids is sufficient to account for all the phenomena of hemiplegia.

Will, in the first place, the course of the decussating fibres account for the production of numbness, or anæsthesia, as well as of muscular palsy; for the former, although not a constant attendant of the latter, may be combined with it? Upon this question no one, who has well examined the anatomy of these parts, will entertain a doubt. The decussating fasciculi of the anterior pyramid, on plunging into the opposite column of the spinal marrow, strike into its centre, and implicate themselves nearly as much with the posterior as with the anterior fasciculi; that is to say, with the sentient as well as with the voluntary portions of the chord; so that the wonder is, not that anæsthesia should be produced through their agency, but that it should be so seldom produced, compared with the frequency of simple muscular palsy.

Again: it is evident that palsy of *all* the spinal nerves of the opposite side of the body may be sufficiently accounted for, as a consequence of lesion of the cerebrum, if the anterior pyramids be supposed to transmit the palsy shock; for the fibres of the pyramids, which extend upwards, on the one hand, into the cerebral hemispheres of the same side, or into the seat of lesion, on the other hand, are continued downwards into the upper part and centre of that tract, from which *the whole of the spinal nerves* are derived. But how is it possible to account for palsy of the opposite side of the face, through the same channel, for palsy of the opposite side of the tongue, and of the opposite facial and auditory nerves? These phenomena may, perhaps, be thus explained: Where the decussating fasciculi of the anterior pyramid plunge into the opposite half of the spinal marrow, they are implicated, in a wonderful closeness of intertexture, with fibres, which, in their upward course, bend towards the places of origin of the ninth and seventh, and of the eighth and fifth of that palsied chord. May it not be supposed that this interlacement may be a sufficient means of communicating the palsying influence to the ascending fibres, which are in close relation to the affected cerebral nerves? Thus, the palsy-stroke transmitted to the junction of the spinal chord and medullary oblongata, might spread its influence in either direction separately, or in both together, according to laws which may possibly be hereafter rigorously determined; sometimes striking the body alone with palsy, sometimes the face, sometimes both; sometimes palsying speech, sometimes deglutition, sometimes hearing.

But the fifth nerve, why is it so rarely affected in hemiplegia? and the orbital nerves, why do they so frequently escape the palsying influence? These phenomena, it will be evident, are highly consistent with the supposition that the palsying force *strikes* exactly at the point where the decussating fibres of the anterior pyramids terminate. Supposing the paralysing impression *to be received* on this part, its force *upwards* should be weakened in proportion to the distance of each cerebral nerve from that part. But the fifth lies further off than the seventh, the third than the fifth; and something in that proportion is the infrequency of the palsy of these nerves in hemiplegia.

But if the phenomena of hemiplegia dependent upon *cerebral lesion* are thus sufficiently explained, how is the fact to be accounted for, that hemiplegia of the opposite side is produced by lesion of one hemisphere of the *cerebellum?* I have little doubt that the following explanation of the phenomenon will eventually be proved to be correct.—The fibres of the anterior pyramids pass through the pons Varolii. The pons Varolii consists in great part of filaments, which issue from each hemisphere of the cerebellum. These filaments may easily be supposed to convey a depressing influence from the diseased hemisphere; but in their course they come immediately upon the filaments of the anterior pyramid of the same side; and they are so implicated with the latter, with such a singular closeness of reticulation, and often with so much that looks like an actual interchange of filament, that it is far from unlikely that they may transmit to the descending fasciculi of the pyramid a shock, which may thence be communicated to the same part at which cerebral lesion exerts its paralysing force.

But why does it not happen that the diseased hemisphere does not strike with palsy the cerebral nerves of the same side, the fifth, the seventh, and the ninth? It is not impossible that the ninth, which rises close to the side of the pyramid, may be so affected. But the fifth and seventh are so remote from the pyramid of the same side, that it could not be expected to extend its influence to them.

But it has been mentioned that there are cases in which hemiplegia of the opposite side to the seat of cerebral affection may coexist with partial palsy of the same side; both phenomena being produced by one cerebral lesion. How are such phenomena to be brought under the same hypothesis? The following case, which is under the care of Dr. Hawkins, in the Middlesex Hospital, and is of remarkable interest, may serve at once to exemplify and to explain the apparent anomalies to which I now advert.

The patient, a female, twenty-eight years of age, (I omit the symptoms of general disorder under which she has laboured,) experienced, in December last, pains deeply seated in the left side of the head; she became afflicted, at the same time, with palsy of the right side of the body. Under appropriate treatment, the pain in the left side of the head has subsided, and the hemiplegia has much diminished. But another symptom supervened soon after the appearance of the hemiplegia: the sight of the left eye has become dim, the pupil is dilated, the upper eyelid has fallen, the eye is more prominent than the other, is habitually directed outwards, and can by no effort be directed inwards. It is evident, from the latter train of symptoms, that the second and third nerves of the left side have become partially palsied. Through palsy of the second, her sight is dim; through the same cause, joined with imperfect palsy of the third, the pupil is dilated; through the palsy of the third, again, the eyelid droops, the levator having feeble action; through the same cause the eye protrudes, for there is no sufficient force in the weakened recti to overcome the traction forward, which is produced by the unpalsied superior oblique. The eye is habitually turned outward through the influence of the fourth and sixth nerves, that are unaffected; and when, by a painful effort, the eye has been slowly drawn to a central position, which the feeble action excited through the *imperfectly* palsied third can gradually effect, the contrast is remarkable of the rapidity with which it is thrown outwards, the moment the patient is desired to look in that direction.

But what is the seat of the lesion which has produced this complicated paralysis? It appears to me impossible to doubt that the disease is situated in or near the left optic thalamus; it has palsied, though not perfectly, the second and third nerves of the same side, which rise in its immediate vicinity; it has then thrown its palsying influence down the fibres of the crus cerebri to the anterior pyramid of the same side; the fibres of the pyramid having no communication with the fourth, the fifth, the sixth, the seventh, or the eighth, of the same side, these have escaped unharmed. Below their origin is the decussation, at which it is certain that the palsy shock has been transmitted to the opposite half of the spinal chord.

What may be the nature of the disease in this case is by no means so certain as its place. Palsy follows various kinds of lesion: sanguineous effusions, softening, abscess, tumours, or pressure from substances external to the meninges of the brain, may frequently produce it. In the present instance, the gradual supervention of the symptoms renders it probable that they are caused either by softening or by the growth of a tumour. The diminution of the hemiplegia under the treatment employed, and the increase of the palsy of the parts in the orbit, lead me to suspect the latter.

3. *What reason can be assigned for the facts, that in total hemiplegia from cerebral lesion, the palsy of the leg is less complete, and capable of being more quickly recovered from, than the palsy of the arm?*

If the hypothesis of a palsy stroke, or paralysing shock, be admitted, it is easy to account for these phenomena, which, it may at the same time be observed, are strikingly irreconcilable with the opposite hypothesis. If a paralysing force be supposed to be transmitted along the decussating fasciculi of one anterior pyramid, as it is natural to expect that where the blow falls, there the effect should be greatest, it is but consistent to expectation that the principal degree of palsy should be produced in the upper part of the spinal marrow, and lower part of the medulla oblongata. Accordingly, nothing is really more frequent than the occurrence of hemiplegia *limited to the face and arm of one side.*

In order to suppress an hemorrhage from the throat, I applied a ligature to the common carotid artery, in a gentleman about thirty years of age: a few days afterwards, palsy of the opposite arm and side of the face supervened. After his death it was found that an abscess had formed upon the right cerebral hemisphere.

A gentleman, eighty years of age, after a day of unusual exertion, in December last, was smitten with palsy: it affected the left arm and the left side of the face. He has now entirely recovered.

A woman was admitted the last week, under the care o Dr. Watson, into the Middlesex Hospital, in a state of insensibility: she gradually regained consciousness, but one arm and the same side of the face were palsied; in a few days she died. A large clot of extravasated blood was found in the substance of the left hemisphere of the brain.

But it is needless to multiply instances of this most frequent occurrence, namely, the combination of hemiplegia of the face and arm of the same side from cerebral lesion of the opposite.

But in the two first cases, to which I have adverted, other features presented themselves: in the first of the two, before death, the leg of the same side became additionally palsied. In the second, the leg of the palsied side was observed to be extremely cold. In both instances, a smaller force of palsy reached the lower limb than had struck the upper.

As a general rule, when hemiplegia slowly invades the entire side, the arm is palsied before the leg. The palsy of the arm is more complete than that of the leg; and, if the patient recovers, the palsy of the leg diminishes, and finally disappears before that of the upper extremity. It is obvious that the hypothesis of a palsy stroke, transmitted by the decussating fasciculi of the anterior pyramid to the junction of the medulla oblongata and spinal marrow, and thence throwing its influence upwards and downwards, (the effect diminishing as the distance in either direction from that point increases,) will account for all these phenomena; which, on the other hand, may fairly be used as strong arguments in proof of the soundness of that hypothesis.

4. *Why, in a slow attack of palsy, are the muscular weakness and the numbness first felt in the extremity of the affected limb? Why in the hand before the fore-arm,—in the fore-arm before the upper arm?*

In this instance it may be presumed that a diminution of the usual quantity of stimulus or energy, transmitted along the nerves from the organs in which they rise, is the cause of the effect observed. A part of the chord is smitten with an imperfect palsy-stroke: it cannot energize as before, or throw along the nerves which arise from it the usual quantity of nervous force. Upon this supposition it would follow that the defect of stimulus should first become sensible at the extremity of a limb. The weakened segment of the chord might be expected to be unable to throw out energy enough to fill a long nerve, which it yet might supply with adequate force of stimulation the shorter nerves of the portion of the limb nearer the trunk. The fact presents a remarkable contrast with the last class of facts adverted to, and shows at all events that the two are not referable to one principle. In slow hemiplegia, the arm is struck before the leg; but the hand is struck before the arm.

5. *How is it to be accounted for that muscular palsy is more frequent than anæsthesia?*

The reason may be this: The office of the sentient nerves is probably an easier function than that of the motor nerves. In some experiments which I made upon the mode and quickness of reparation of both classes of nerves after their division, I found that the sentient nerves resumed their functions in a shorter time than the voluntary nerves. I divided the facial branches of the seventh and of the fifth nerves, on one side in a cat: in fifteen days sensation had returned, but no sign of returning motion appeared till the twenty-first. If this principle be true, it will solve the present question. The sentient nerves, it would appear, require a harder blow to palsy them than the voluntary. It deserves besides to be pointed out, that, as the office of the former is to transmit towards the centre, not from the centre, it is natural to expect that they would be less susceptible of a force proceeding against the habitual course of these motions. The connexion of the decussating fibres of the pyramid with the posterior part of the opposite half of the spinal chord, is likewise not *quite* as extensive as with the anterior.

The medulla oblongata, to which so much importance has been attached in the preceding observations, has on other grounds the greatest interest of any part in the whole economy: by a strange property, although its direct office, as far as the mental operations are concerned, is probably subordinate, it is nevertheless the immediate link which binds us to life; so that all the endowments of the mind are at once corporeally extinct, and the soul liberated from its tenement, when this small organ is mutilated. Perhaps it is more wonderful still, that this small and central part can actually determine the persistence of consciousness in either portion of a divided animal: it determines, for instance, in a cold-blooded animal, whether feeling and volition shall persist in the body or in the head, and perish in the other. In decapitating a turtle, if the section be made below the medulla oblongata, the body is instantaneously dead, but the head remains alive for hours. If, on the other hand, the section be made half an inch higher, the head is instantaneously bereft of life; the body, for days afterwards, remaining alive, and endued with feeling. Nor is it through the whole medulla oblongata, small as that part is, that this vitalizing property is diffused: it can be proved to be confined to a small portion of its upper end; the part, namely, at which the fifth, seventh, and eighth nerves take their rise. In frogs and turtles, which possess such a power of surviving common mutilation, if, every other part remaining entire, this little segment be deeply injured with the finest point, the limbs, and every part, are at once relaxed in death, and life is utterly and instantaneously extinguished.

One might almost fancy that this striking phenomenon had been known to our great Poet, from a passage which I will venture to quote, in concluding the present remarks, so curiously true to Nature is the following imagery: yet is it no more than one additional proof to many of that intuitive justness of fancy and penetrative imagination with which the mighty Mother had endued her favoured son.

"Within the hollow crown
That rounds the mortal temples of a king,
Keeps Death his court; and there the antic sits,
Scoffing his state, and grinning at his pomp;
Allowing him a breath, a little scene
To monarchize, be feared, and kill with looks;
Infusing him with self and vain conceit,
As if the flesh that walls our mortal life
Were brass impregnable;—till, humoured thus,
He comes at last, and with a little pin
Bores through his castle wall, and farewell king."

*To the Editor of the Medical Quarterly Review.*

SIR: Should the following case, with the accompanying remarks, illustrated by specimens from my own morbid anatomical collection, be considered sufficiently interesting to merit a place in the Medical Quarterly Review, they are very much at your service.

I remain, Sir, your obedient servant,

JOHN HOWSHIP.

*Saville Row; January* 16, 1835.

*References to the Figure.*

A. The shaft of the right femur.
B. The external condyle.
C. The internal condyle.
D. The large excavated opening, the external margin of which, at this part, demonstrates the outer surface of the femur, thin as paper.
E. The exposed cavity of one of the enlarged longitudinal canals.
F. The thin cartilaginous surface of the internal condyle.
G. A mass of fine light cancellated structure remaining within the articulating surface of the condyle.

## Figures and Tables

**Figure f1:**